# Burns

**Published:** 1867-12

**Authors:** 


GLYCERINE,

BURNS, whether in children or adults, giving an almost instantaneous relief from suffering, by its entire exclusion of the air and by its moistening, hence cooling, soothing effects, promotes a speedy healing process, always safe, simple and efficient. A few cents will buy half a pound of it at any good drug store, and every family should have some at hand, in a bottle, plainly labelled, with a bottle of glycerine at its side.

If glycerine alone is applied with a common brush to the surface of the throat in that dangerous malady dyptheria, in a few minutes its permeative quality enables it to sink into and between the molecules of the false membrane, dissolving and than detaching it, in a few hours; so also in that other most alarming malady, the croup of little children, as it not only penetrates and thus disintegrates, spreads apart the tissues of the false membrane, but when it gets to the bottom of it, it spreads out between the layer of the membrane and the more natural mucous surfaces beneath and thus not only detaches the membrane, but imparts a healing, soothing influence to the inflamed condition of the natural surfaces. Hence also if applied to

